# Harnessing nonlinear rubber swelling for bulk synthesis of anisotropic hybrid nanoparticles†
†Electronic supplementary information (ESI) available: The TEM images of control Au–PDVB hybrid nanoparticles formed without the presence of PVP and Au–PS hybrid nanoparticles. See DOI: 10.1039/c4tc01660b



**DOI:** 10.1039/c4tc01660b

**Published:** 2014-09-19

**Authors:** Tao Ding, Stoyan K. Smoukov, Jeremy J. Baumberg

**Affiliations:** a Department of Materials Science and Metallurgy , University of Cambridge , 27 Charles Babbage Road , Cambridge CB3 0FS , UK . Email: sks46@cam.ac.uk; b Nanophotonics Centre , Cavendish Laboratory , University of Cambridge , CB3 0HE , UK . Email: dt413@cam.ac.uk

## Abstract

Asymmetric hybrid nanoparticles are at the forefront of colloidal chemistry as building blocks for novel structures and applications, as well as for exploring fundamental ways of breaking symmetry in physical systems. Current methods of synthesis have significant limitations in terms of control over synthesis, particle size ranges and polydispersity. We report a facile and scalable synthesis based on the anisotropic swelling of rubber to obtain metal–(polymer rubber) hybrid nanoparticles. Initial Au nanoparticle (NP) seeds are grown larger by reducing HAuCl_4_ with divinyl benzene (DVB), while simultaneous radical polymerization of DVB forms a cross-linked rubber layer of PDVB on the Au NP surface. The propensity of rubber to swell nonlinearly in the presence of DVB monomers amplifies initial asymmetries to break the symmetry of the PDVB shell, causing growth of asymmetric protrusions on one side of the core–shell particles, which are fixed by further polymerization. Plasmonic absorption of Au allows us to follow the Au reduction reaction and also suggests potential applications of some of the asymmetric particles in plasmon-enhanced sensing. The polydispersity, determined statistically from TEM and SEM images, of the resulting particles is low (<10%) and their sizes, shapes and metal–polymer ratios are easily tunable.

## Abstract 2

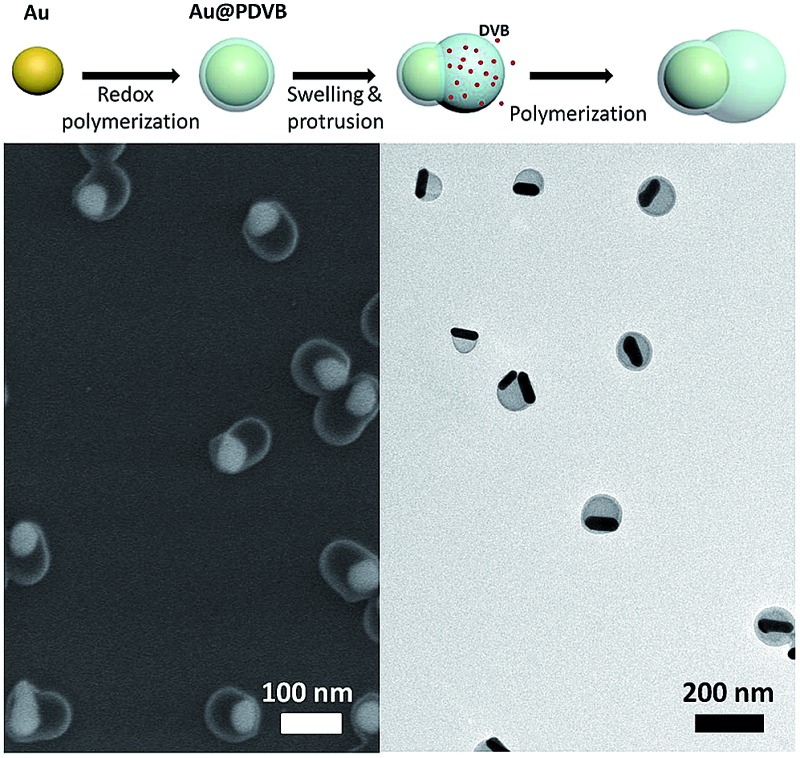
Facile, scalable synthesis of monodisperse anisotropic hybrid nanoparticles (Au nanospheres/nanorod cores and poly(divinyl benzene) shell). Mechanism is based on nonlinear swelling of the polymer during a seeded polymerization process.

## Introduction

Anisotropic hybrid colloidal particles have aroused interest in the last few years for use in multi-functional diagnostics and therapeutics,^1^ catalysis,^2,3^ with potential as solid stabilizers in foams and emulsions,^4^ and suitability for novel self-assemblies of multi-configurations and multi-functionalities that cannot be easily achieved from spherical building blocks.^5–9^ In the last decade, methods for formation of anisotropic hybrid particles have been greatly developed and can generally be classified into physical fabrication and chemical synthesis approaches. Physical fabrication techniques involve lithography,^10^ sputter deposition,^11^ electric field co-jetting,^12^ and microfluidics.^13^ They produce relatively large nanoparticles, but at the same time lack volume scalability. Chemical approaches overcome the volume scaling limitation, but often produce mostly spherical shapes since interfacial energies favor the lowest surface-to-volume ratio shapes. Additional potentials need to be applied to favor (thermodynamically or kinetically) nonspherical shapes. Facet control^14^ and ligand control^15^ have been successfully applied to inorganic composite nanoparticles, however, for organic/inorganic hybrid nanoparticles, the main challenge still remains.

One way to affect the shape of hybrid colloids is hydrophilic–hydrophobic ligand segregation on the surface of metal particles so that the diblock polymers can be self-assembled on the hydrophobic domains.^16^ The controllability of the sizes of the polymer component is poor, however, and limited to a small range of thicknesses from 10 to 20 nm. A more robust and scalable way to create hybrid asymmetric nanoparticles is to initiate polymerization on inorganic nanoparticles or their precursors. The group of Xia introduced a scalable method by initiating precipitation polymerization of PS in the presence of Au NPs.^17^ However subtle effects from the addition time (∼2 s after initiating the polymerization) and sensitivity to reactant ratios make this method difficult in terms of reproducibility. Hybrid nanoparticles have also been prepared by interfacial polymerization,^18^ but reactions at interfaces also require delicate control, and occasionally sophisticated manipulation of one of the suspending phases.

Seeded polymerization is a versatile method for generating nonspherical polymer–polymer hybrid particles. The shapes and ratios of the particles are tunable, driven by the phase separation of polymers.^19–21^ This technique has also been used to create hybrid metal–polymer particles *via* partial wetting of the metal particles before polymerization, though the surface wetting properties are not easy to control.^22^ Polydispersity is also significant, controlled by an Ostwald ripening mechanism.

New methods that are easier to control and which would expand the range of polymers and metals used are highly desired in this field. Here we initiate polymerization of divinyl benzene (DVB) on AuNP seeds to generate a cross-linked rubber coating, but a similar procedure could be used with most rubber coating materials. We use non-uniform swelling of the rubber by its liquid monomer to break the symmetry and form hybrid particles of AuNPs anisotropically encapsulated in poly(divinylbenzene) (PDVB).

## Results and discussion

**Scheme 1:**
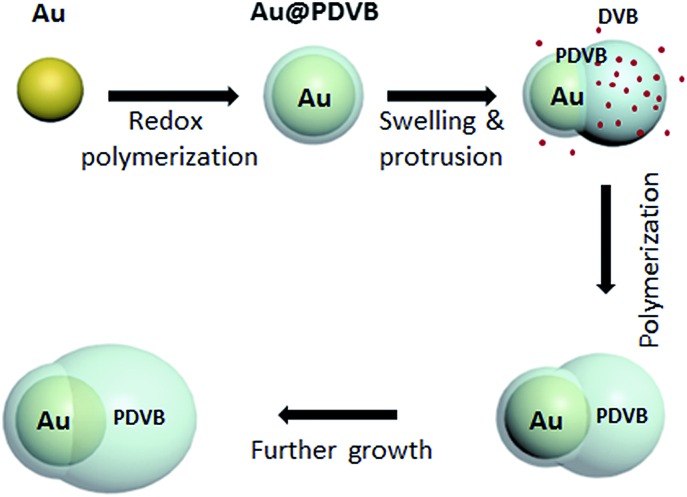
Formation of anisotropic hybrid nanoparticles made of Au–PDVB. The yellow sphere is the AuNP and the red dots represent DVB monomers, which swell, crosslink and enlarge the light blue region representing the PDVB coating.

The synthesis of asymmetric hybrid nanoparticles is based on a combination of seeded polymerization and dispersion polymerization. The detailed growth mechanism is shown in Scheme 1. We used citrate stabilized Au NPs as the seeds. The redox reaction between HAuCl_4_ and DVB will preferentially deposit the reduced Au(0) onto the Au NP seeds along with a uniform coating of PDVB on the surface. The solubility parameter of PDVB is 9.1, which matches quite well with DVB (*δ* = 8.5)^23^ and makes DVB a good solvent/swelling agent for it, as well as its second function as a cross-linker. DVB would swell/uncoil any regions between cross-links until the swelling pressure is balanced by the elastic stress from the network, but the reaction process proceeds in parallel. The elasticity of a network (and its resistance to swelling) is directly proportional to its crosslink density. So any initial asymmetries in swelling are later amplified, since DVB would swell new lightly cross-linked regions more than heavily cross-linked regions. In this paper, we have aimed to produce a simple synthetic procedure, but if one is willing to separate the swelling and reaction processes, one could conceivably control these effects using Flory and Rehner's theory to measure these formalized interactions.^24,25^ Such control on a larger scale was recently used to produce asymmetric particles from two polymers (PS and PDVB)^20^ and could be an inspiration for the development of more complex nanoparticles as well.

**Fig. 1:**
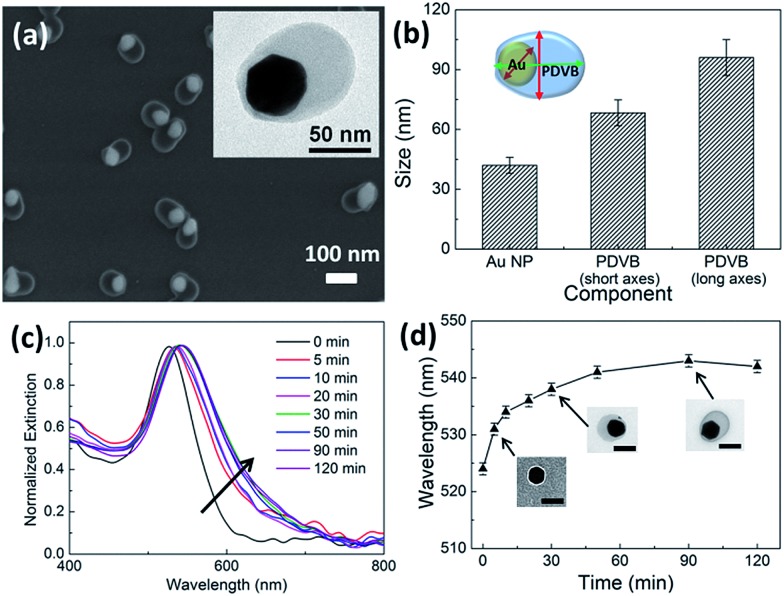
Seeded growth of asymmetric hybrid Au–PDVB colloids. (a) SEM image of the hybrid hetero-dimers with the inset of the corresponding TEM image and (b) histogram of the sizes of different components of the hybrid Au–PDVB nanoparticles (50 particles). The inset shows the way the sizes were measured, Au NPs: dark red arrow; short axes: red arrow; long axes: green arrow. (c) UV-vis extinction spectra of the hybrid Au–PDVB particles measured at different reaction times, and (d) wavelength shift of the peaks in (c) with time, with insets showing TEM images of Au–PDVB hybrid nanoparticles evolved from spherical core–shell to anisotropic encapsulation. Scale bars are 50 nm.

In our case, the swelling of low crosslink density regions results in breaking of the spherical PDVB shell symmetry to form a DVB-swollen lobe.^19^ The swollen rubber lobes of particles continue to swell in a self-catalytic process, as they keep growing and crosslinking, while preserving the particle asymmetry. At the same time, the reaction rates allow us, by the amount of DVB and Au salt added, to tune the ratio between metal and polymer components. Fig. 1a shows SEM and TEM images of the colloids. For a typical reaction, 0.5 ml aqueous solution of Au seeds is sequentially mixed with 40 μl PVP (20 mg ml^–1^), 1 μl DVB and 30 μl HAuCl_4_ (10 mM) and sonicated for 2 h. After this, 50 particle images in SEM were recorded to quantitate their anisotropy and polydispersity, summarized in Fig. 1b. The diameter of the Au component increases from 32 nm (for the initial seeds) to 45 nm. The long and short axes of the PDVB ellipsoids are 68 and 96 nm with a relative standard deviation of 10%. The reaction kinetics was monitored by UV-vis spectroscopy as shown in Fig. 1c and d. The TEM image at the initial stage of growth (5 min reaction) shows that ∼3 nm of PDVB is wrapped on the surface of Au nanoparticles to form a concentric core–shell structure (see the left inset in Fig. 1d). As the polymerization proceeds, the sizes of both the Au NP and the PDVB components increase, but the encapsulation evolves quite eccentrically due to the nonlinear swelling of PDVB as shown in the middle and right insets in Fig. 1d. The reaction usually finishes in just over an hour, when the plasmonic absorption peak has red-shifted from 524 to 543 nm, which is mainly due to the increase of the size of Au NPs.

We use water as the dispersant medium and PVP as the stabilization agent. Assuming full coverage of the resulting particles, it is estimated from the amount of PVP added that around 10^5^ PVP molecules were adsorbed on the surface of the Au–PDVB nanoparticles. Without PVP, the hybrid nanoparticles of Au–PDVB aggregate (see ESI-Fig. S1†). Cross-linking is critical for the generation of anisotropic nanoparticles since if styrene (with only one vinyl bond) is used as the monomer, only a very thin layer of PS shells is coated on the surface of the Au NPs and no protrusion is observed (see ESI-Fig. S2†). It is possible that the mismatch of interfacial energy between PDVB and Au NPs may also cause anisotropic growth, but this would be more likely to produce hetero-dimers rather than ellipsoids.^22^

**Fig. 2:**
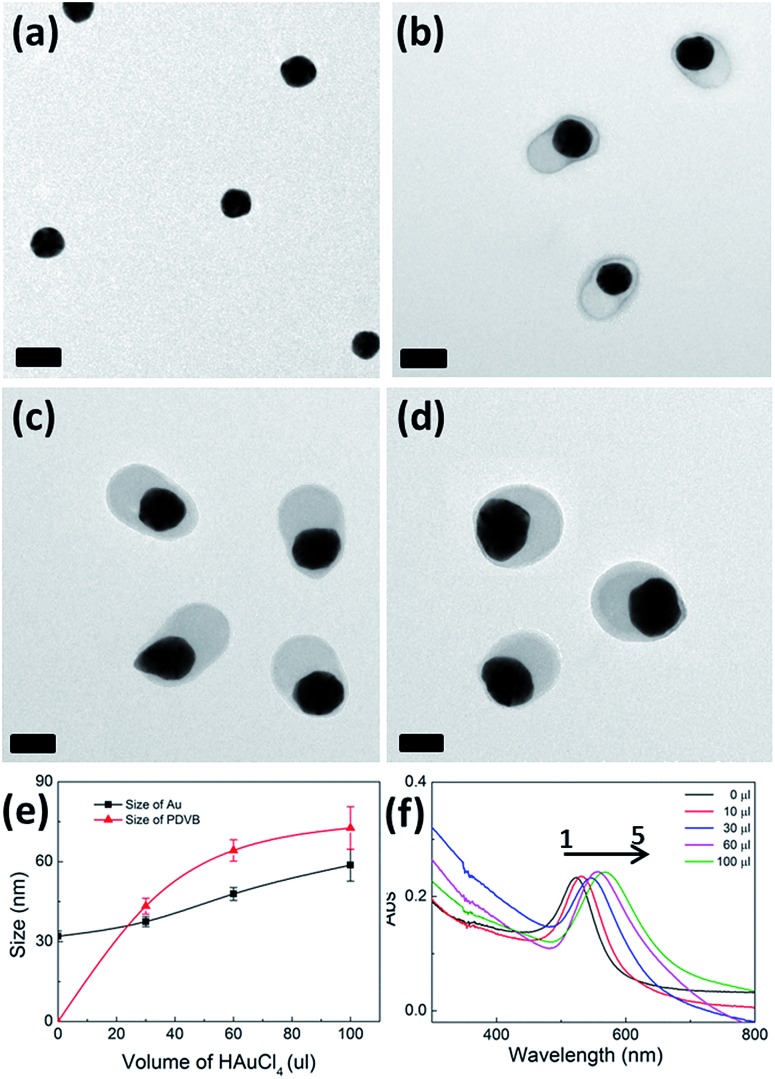
Increasing size of hybrid Au–PDVB hetero-dimers with increasing amounts of HAuCl_4_. (a–d) TEM images with increasing 10 mM HAuCl_4_ from (a) 0 μl, (b) 30 μl, (c) 60 μl, to (d) 100 μl, with fixed 1 μl DVB. Scale bars are 100 nm. (e) Measured size of Au and PDVB particles (short axis) with increasing HAuCl_4_. (f) Corresponding UV-vis absorption spectra of the Au–PDVB hetero-dimer nanoparticles. Spectra 1 to 5 correspond to particles with increasing HAuCl_4_ added, (1) 0 μl, (2) 10 μl, (3) 30 μl, (4) 60 μl, and (5) 100 μl.

By adjusting the amount of HAuCl_4_, the absolute size of the Au NP cores can be controlled independently (Fig. 2). We start with 32 nm diameter Au NP seeds and vary the amount of HAuCl_4_ added while keeping the same amount of DVB. With increasing amounts of HAuCl_4_, the size of the Au NP increases gradually, as does also the size of the PDVB shell, since HAuCl_4_ also initiates conversion of DVB in solution to PDVB. The PDVB shell size gradually reaches a plateau beyond 100 μl HAuCl_4_, implying that full conversion from the DVB monomers to PDVB has occurred. The hybrid nanoparticles maintain the same asymmetric shape throughout the growth process as shown in Fig. 2b–d. The statistical range of sizes of the Au and PDVB components in the hybrid nanoparticles is shown in Fig. 2e, with polydispersity remaining small. The plasmonic properties of the hybrid nanoparticles are characterized by UV-vis spectroscopy (Fig. 2f), showing the absorption peak red-shifts from 524 nm to 567 nm with increasing amounts of HAuCl_4_. At the same time, the spectral half width of the plasmonic peak increases considerably from 60 to 100 nm. These measurements can be explained by both the increase in the size of the Au NPs, as well as the increase in the effective refractive index surrounding them. We note that such solution measurements average over the anisotropy of the hybrid NPs, combining plasmons across the short axis with those along the long axis (which experience more of the higher refractive index PDVB), thus broadening the measured linewidth.

**Fig. 3:**
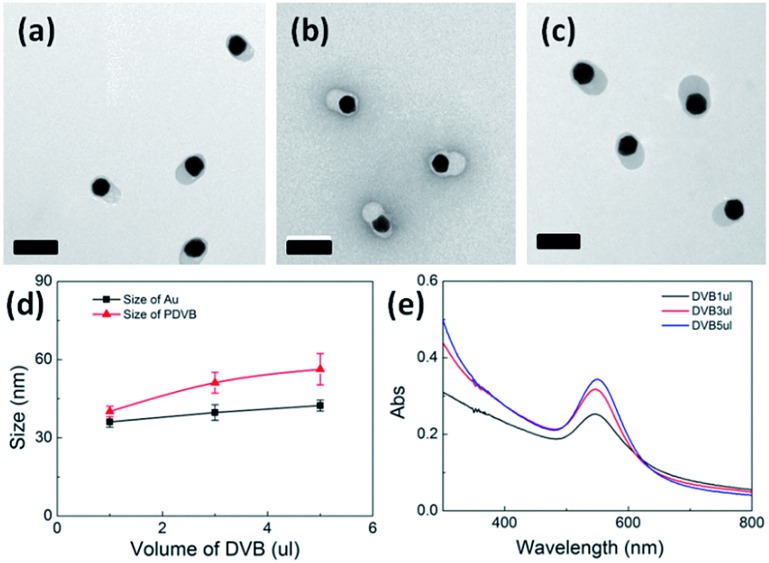
Size of hybrid Au–PDVB hetero-dimers with increasing DVB of (a) 1 μl, (b) 3 μl, and (c) 5 μl, while keeping 0.6 mM concentration of HAuCl_4_ fixed. Scale bars are 100 nm. (d) Size dependence of Au and DVB components of hybrid nanoparticles *vs.* DVB volume, with (e) corresponding absorption spectra.

We also examined the size change with increasing volumes of DVB from 1 μl to 5 μl, while keeping the amount of HAuCl_4_ fixed (Fig. 3). The PDVB particle size increases gradually from 40 to 50 nm, while the size of Au NPs increases only slightly from 32 to 37 nm (Fig. 3a–d), perhaps due to a higher conversion ratio of Au(i) to Au(0) with excess reductant (DVB). In all cases, the amount of DVB is in excess and the amount of HAuCl_4_ is the limiting factor for the growth of both PDVB and Au NP components. The absorption spectra of the plasmonic nanoparticles (Fig. 3e) show a small peak shift, confirming our previous hypothesis that the peaks red-shift mainly due to increasing Au NP size, with less dependence on the PDVB shell size.

We normally carried out the experiment near 45 °C. The process is one of the seeded-growth methods, so small temperature variations should not change the final particle size, which is determined by the exhaustion of the reactants (HAuCl_4_) and the DVB monomer used. The kinetics of the process may change but the number of Au and Au–PDVB hybrid nanoparticles remains constant.

**Fig. 4:**
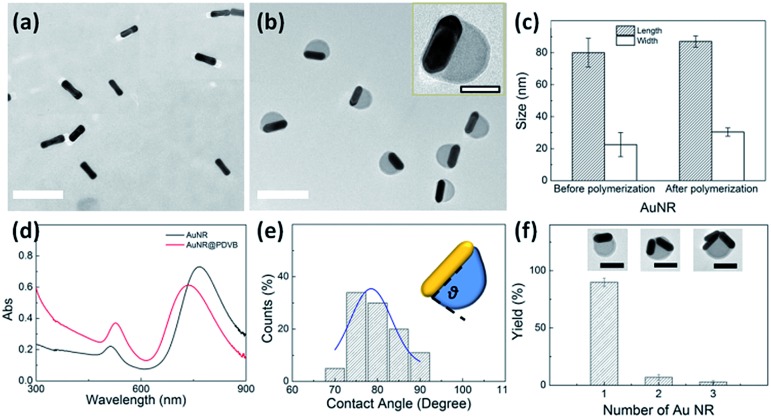
Growth of asymmetric hybrid nanoparticles using Au NR seeds. (a) Seed Au NRs, and (b) as-grown asymmetric AuNR–PDVB hybrid nanoparticles. Scale bars are 200 nm and 50 nm in the inset. (c) Statistical distribution of Au NR dimensions before and after growth of asymmetric AuNR–PDVB hybrid nanoparticles. (d) Absorption spectra of Au NRs and AuNR–PDVB hybrid nanoparticles. (e) Statistical distribution of the observed angle between PDVB and AuNRs, with Gaussian fit (blue line). (f) Yields of anisotropic hybrid nanoparticles with different numbers of AuNRs encapsulated.

In order to increase the asymmetry of the hybrid particles, we attempted to use Au NRs as the seeds (Fig. 4a) on which the PDVB shell is grown. Two options are then possible: (a) that one side would start swelling more and thus dominate with growth on just one side of the rods or (b) that if the two sides are sufficiently separated, both may grow independently. In fact (Fig. 4b), the Au NRs grew PDVB lobes exclusively on just one side. Partly this may be because the high curvature of the Au NRs prevents complete wetting of the rods with DVB, and only the side initially wetted grows, since in the presence of swellable PDVB no further nucleation or wetting of DVB on Au takes place. Statistical measurements of the Au NR (50 particles) sizes (Fig. 4c) show that their length increases (from 80 to 87 nm) and their width increases (from 23 to 30 nm), implying that deposition of Au(0) on the NRs is uniform all over the surface. The NR aspect ratio thus reduces from 3.5 to 3.0, which in the extinction spectra of the hybrid AuNR–PDVB nanoparticles (Fig. 4d) produces a blue-shift of the longitudinal mode (from 769 to 731 nm) and a red-shift of the transverse mode (from 513 to 526 nm) compared to the original Au NR seeds.^26,27^ The basic mechanism appears to be the same as with Au NP seeds. The reduced Au(0) heterogeneously grows on the Au NRs along with a thin layer of PDVB oligomers formed at the surface. The DVB monomer droplets partially wet the Au NR surface and grow anisotropically on one side. The contact angle between PDVB and Au NR becomes consistently ∼80° (Fig. 4e). Although the number of Au NRs on the PDVB lobe can vary up to three (Fig. 4f, insets), the majority of products are AuNR–PDVB particles with a single rod (90%). Particles with two (8%) or three rods (2%) may possibly be generated at the initial stage of the dispersion polymerization when the DVB lobe is not totally solidified and occasional collisions can occur between the particles. The high stabilization from PVP molecules prevents much aggregation, resulting in the near-uniform monodisperse distribution of rods in particles.

The near hemispherical shape of these hybrid nanoparticles containing a single rod is interesting from both assembly and sensing perspectives. The almost complete absence of the polymer on one side of the particles implies large differences in polarization, which has been used before in the assembly of similar Janus particles in electric^28^ and magnetic fields.^29^ Furthermore, when the lobes are grown to larger diameters, due to their size and symmetry, the particles would only be able to assemble in pairs (when aggregated by electric or optical fields), since the sideways hemispherical protrusion would prevent a third particle from coming in contact with the rods. Knowing only two particle cores could interact, this polarization enhancement could be used for quantitative and sensitive detection. This is a unique advantage in suspensions, as traditionally such an enhancement has been possible only through the laborious fabrication of nanolithography patterns,^30^ and has long been sought after in the self-assembly of plasmonic structures.^31^

## Experimental

The Au seeds (AuNPs and Au NRs) were obtained from Sigma-Aldrich and used as received. Typically, 40 μl of PVP (20 mg ml^–1^) was mixed with 0.5 ml of the Au aqueous solution in a 1.5 ml Eppendorf tube. The aqueous solution of HAuCl_4_ (10 mM) was introduced into the mixture under vortex mixing followed by the addition of the monomer of DVB. The amounts of HAuCl_4_ and DVB were adjusted to tune the relative sizes of Au and PDVB in the hybrid nanoparticles. The entire Eppendorf tube was kept in an ultrasonic bath (600 W, 45 °C) for 2 h. The final products of Au/PDVB asymmetric hybrid nanoparticles were harvested after 3 cycles of centrifugation and redispersion.

The morphology of asymmetric hybrid nanoparticles was characterized using a Scanning Electron Microscope (Zeiss) and a Transmission Electron Microscope (FEI Tecnai 20). For clear observation of the polymers, some of the samples were stained with (NH_4_)_6_Mo_7_O_24_. The plasmonic properties of the hybrid nanoparticles were characterized using a UV-vis spectrometer (Ocean optics QE65000).

## Conclusions

In this paper, we report a facile approach for asymmetric hybrid plasmonic nanoparticles made of Au and PDVB. This synthetic approach is easy to control and superior to physical approaches in terms of scalability, control of small size, and uniformity. Specifically, we synthesized hybrid nanoparticles of Au and PDVB rubber. After initial formation of the thin rubber shell, nonlinear swelling by the monomer in suspension causes a self-amplifying bulge on one side of the Au particle, which continues to grow as long as there is a monomer in the suspension. By adjusting the size of the initial seeds, the amount of Au precursor added, and the amount of DVB monomer, we can tune the size of the Au core and the polymer shell and achieve good monodispersity. The hybrid particles are stable and do not easily aggregate, due to the lower van der Waals interactions of their polymer shells, which make them good for plasmonic probes. We can fine-tune the plasmon resonance wavelength of the cores to match specific requirements.

The strategy we have used here is not only applicable to Au and DVB, but also relies on the non-linear swelling of the rubber shell. It should thus be possible to use it with any cross-linked rubber shell material which is swollen by its liquid monomer. Similarly, the Au core could be replaced by another metal, which, for Ag or Cu, could also exhibit plasmonic properties, but for other metals may at least be protected from the environment by the polymer shell.

Potential applications for the hybrid plasmonic particles include sensing, which may be especially true for the hybrid particles starting from Au NRs, which we have demonstrated. Including magnetic cores could allow various particle assemblies in the presence of electric or magnetic fields. We hope the fundamental insight of breaking symmetry for hybrid particles through rubber swelling, the scalability of this dispersion method, its ease of use, and low polydispersity will enable the synthesis of a whole class of such particles.
