# Supplementary material for: Harnessing nonlinear rubber swelling for bulk synthesis of anisotropic hybrid nanoparticles
Source: J Mater Chem C Mater. 2014 Sep 19;2(41):8745–9. doi: 10.1039/c4tc01660b (PMC4894071; doi:10.1039/c4tc01660b)
Supplement: Supplementary file 1 [file TC-002-C4TC01660B-s001.pdf]

Electronic Supplementary Material (ESI) for Journal of Materials Chemistry C.
This journal is © The Royal Society of Chemistry 2014

Supporting Information

Harnessing Nonlinear Rubber Swelling for Bulk Synthesis of Anisotropic Hybrid Nanoparticles

Tao Ding,*[ab] Stoyan K. Smoukov,*[a] Jeremy J. Baumberg[b]

[†]Department of Materials Science and Metallurgy, University of Cambridge, 27 Charles Babbage Road, Cambridge CB3 0FS, UK. [‡]Nanophotonics Centre, Cavendish Laboratory, University of Cambridge, CB3 0HE, UK

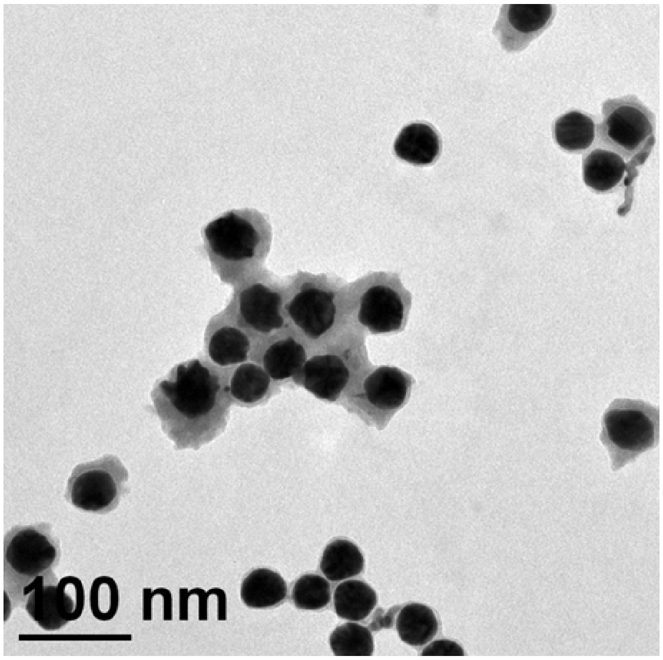

Figure S1. With Au NPs as the seeds, but with no PVP in presence, the PDVB were aggregated along with Au NPs.

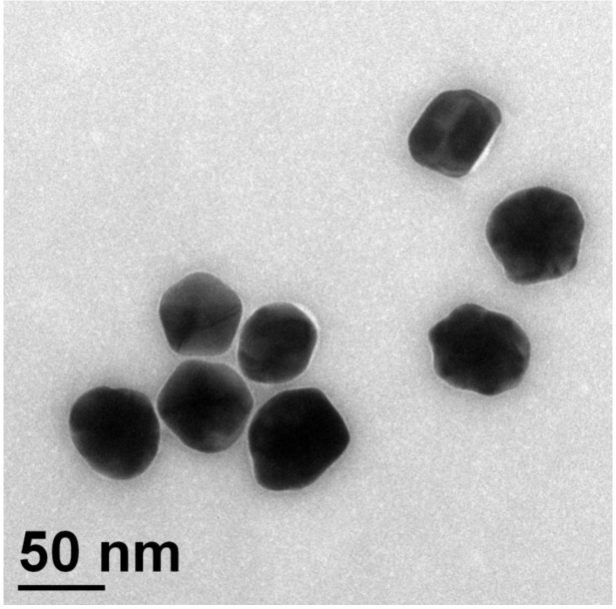

Figure S2. The TEM image of Au-PS hybrid nanoparticles obtained with styrene as the monomer.
